# Comparison of HER2 status in primary and paired metastatic sites of gastric carcinoma

**DOI:** 10.1038/bjc.2011.121

**Published:** 2011-04-12

**Authors:** C Bozzetti, F V Negri, C A Lagrasta, P Crafa, C Bassano, I Tamagnini, G Gardini, R Nizzoli, F Leonardi, D Gasparro, R Camisa, S Capelli, E M Silini, A Ardizzoni

**Affiliations:** 1 Medical Oncology Unit, Azienda Ospedaliero-Universitaria, Via Gramsci 14, Parma 43126, Italy; 2 Pathology Department, Azienda Ospedaliero-Universitaria and Centro di Oncologia Molecolare Traslazionale, Università, Parma, Italy; 3 Pathology Department, Arcispedale ‘S Maria Nuova’, Reggio Emilia, Italy

**Keywords:** HER2, gastric cancer, FISH, immunohistochemistry

## Abstract

**Background:**

Trastuzumab has recently shown efficacy in the treatment of HER2-positive advanced gastric adenocarcinoma. Although antibody-based therapies target the metastatic disease, HER2 status is usually evaluated in the primary tumour because metastatic sites are rarely biopsied. The aim of this study was to compare HER2 status in primary and paired metastatic sites of gastric adenocarcinoma.

**Methods:**

The HER2 status was assessed by fluorescence *in situ* hybridisation (FISH) and immunohistochemistry (IHC) in 72 secondary lesions of gastric adenocarcinoma and in the corresponding primary tumours.

**Results:**

Concordance of FISH results, evaluable in 68 primary and matched metastatic sites, was 98.5%. Concordance of IHC results, available in 39 of the 72 paired cases, was 94.9%. Only one case showed discordance between primary tumour and metastasis, being negative by both IHC and FISH in the primary and showing HER2 overexpression and amplification in the corresponding pancreatic lymph node metastasis.

**Conclusion:**

The high concordance observed between HER2 results obtained by both IHC and FISH on primary tumours and corresponding metastases suggests that in gastric cancer HER2 status is maintained in most cases unchanged during the metastatic process.

Trastuzumab in combination with chemotherapy can be considered as a new standard option for patients with HER2-positive advanced gastric or gastroesophageal junction cancer (Bang *et al*, 2010). In gastric adenocarcinoma, HER2 gene amplification or protein overexpression is found in 7–34% of primary tumours (Takehana *et al*, 2002; Tanner *et al*, 2005; Park *et al*, 2006; Gravalos and Jimeno, 2008; Hofmann *et al*, 2008; Barros-Silva *et al*, 2009; Marx *et al*, 2009). Although trastuzumab-based therapy is used to treat metastatic disease, HER2 status is generally evaluated in the primary lesions because metastatic sites are rarely removed or biopsied before treatment. However, it is still unknown whether HER2 status differs in metastases compared with primary tumours. To date, only one study has addressed this issue showing a high level of concordance between HER2 status evaluated on primary tumours and regional lymph node metastases by means of both immunohistochemistry (IHC) and fluorescence *in situ* hybridisation (FISH; Marx *et al*, 2009).

In this study, HER2 status was assessed by FISH and IHC on samples obtained from metastatic sites of gastric carcinomas and paired primary tumours. The aim of the study was establishing whether HER2 status assessed on primaries could be reliable for treatment decisions with anti-HER2 agents in patients with metastatic gastric cancer.

## Materials and methods

The current series included 72 consecutive patients with gastric adenocarcinoma who underwent diagnostic fine-needle aspiration biopsy (FNAB) or tissue biopsy or surgical resection of synchronous or metachronous metastatic lesions. Tissue specimens of the paired primary tumours (18 endoscopic biopsies and 54 gastric resections) were available for all patients. The *HER2* status was tested by FISH in both cytological and histological samples. The *HER2* FISH results were confirmed by IHC on available histological sections. None of the patients were treated with trastuzumab-based regimens.

### Cytological specimens

Cytological smears from metastatic lesions were obtained by multidirectional ultrasound-guided FNAB using a 22-gauge for deep lesion and 22–23 gauge for superficial lesions. The aspirated material was smeared on glass slides and air dried. Cellular suspensions obtained from pleural and ascitic fluids were cytocentrifuged and air dried. At least two slides were stained with May–Grünwald–Giemsa for routine cytology. The remaining slides were kept unstained at −20°C until assay. After cytological diagnosis of malignancy, one representative slide was submitted to *HER2* FISH.

### Histological specimens

Formalin-fixed, paraffin-embedded tissue blocks, selected on the basis of quality and representativeness of the sample, were cut into 3-*μ*m thick sections that were submitted to both FISH and IHC. For each case, two tissue sections cut at different levels of the histological block were processed to control for tissue heterogeneity.

### *HER2* assessment – FISH

The *HER2* amplification was assessed on both histological and cytological samples using a Spectrum Green fluorophore-labeled *α*-satellite DNA probe for chromosome 17 (Chr17) and a Spectrum Orange fluorophore-labeled DNA probe for the *HER2* gene locus (Vysis PathVysion HER-2 DNA Probe Kit, Vysis-Abbott, Wiesbaden, Germany). Slides were hybridised using a Hybrite denaturation/hybridisation system for FISH (Vysis). Details of the method were previously described (Bozzetti *et al*, 2003). The FISH images were processed utilising an Olympus MX60 fluorescence microscope (Olympus, Hamburg, Germany) with a 100-W mercury lamp. Separate narrow band pass filters were used for the detection of spectrum orange, spectrum green and DAPI. At least 100 evaluable nuclei for each case were scored visually in different tumour areas by two independent observers in blind (CB, CL) giving superimposable results. Cases showing two Chr17 in >50% of cells were classified as disomic. Chromosome 17 polysomy was defined as ⩾3 *CEP17* signals on average per cell. Amplification was defined as a *HER2*/*CEP17* ratio ⩾2, or when an *HER2* signal cluster was observed (Hofmann *et al*, 2008).

### HER2 assessment – IHC

Sections of archival formalin-fixed, paraffin-embedded tissue (3 *μ*m) were placed on slides coated with poly-L-lysine. After deparaffinisation and blocking of endogenous peroxidase, HER2 immunostaining was performed using rabbit anti-human c-erbB-2 oncoprotein as primary antibody (Dako Corp, Carpinteria, CA, USA) at 1/100 dilution. Binding of the primary antibody was revealed by means of the Dako Quick-Staining, Labelled Streptavidin–Biotin System (Dako), followed by the addition of diaminobenzidine as a chromogen.

The HER2 immunoreactivity was evaluated by an experienced pathologist according to the scoring system of Hofmann *et al* (2008). Resection samples exibiting a strong (3+) complete, basolateral or lateral membranous reactivity in ⩾10% of the cells were scored as positive. Samples with no reactivity or membranous reactivity in <10% or faint or barely perceptible membranous reactivity (1+) in ⩾10% of tumour cells (cells are reactive only in part of their membrane) were considered negative. Samples showing a weak to moderate complete, basolateral or lateral membranous reactivity (2+) of ⩾10% of tumour cells were scored as equivocal. For tumour biopsy specimens the same patterns were considered, but irrespective of the percentage of tumour cells.

### Statistics

Pearson's correlation test was used to compare the HER2 status assessed by IHC and FISH on metastatic sites. A strong correlation was defined as a correlation coefficient *r*⩾0.8.

Concordance between IHC status on primary *vs* metastatic sites was calculated as the ratio of concordant cases to total cases. The *κ*-coefficient was used to assess the level of agreement between samples. The *κ*-values ranging from 0.61 to 0.8 were assumed to indicate a very good agreement; *P*<0.05 allowed us to reject the null hypothesis that there is no agreement.

Differences were considered statistically significant when the *P*-value was ⩽0.05. All statistical tests were two-sided.

## Results

The *HER2* gene copy number was evaluated by FISH in 72 consecutive primary gastric adenocarcinomas (18 biopsies and 54 resection specimens) and their corresponding metastatic lesions (33 FNAB samples, 9 core tissue biopsies and 30 surgical resections). The main characteristics of the patients are summarised in Table 1. Secondary lesions were localised to liver (*n*=19), pleura (*n*=4), peritoneum (*n*=19), skin (*n*=4), regional lymph nodes (*n*=2), distant lymph node (*n*=14) and 10 to other sites. Of the 33 metastatic lesions sampled by cytology, 79% (26/33) were metachronous compared with 26% (10/39) of those sampled by surgery. In all, 4 of the 72 primary tumour specimens were not suitable for *HER2* status assessment because of the poor fixation of tissue, whereas all specimens from metastatic sites were adequate for evaluation.

Among the metastatic lesions, *HER2* amplification was observed in 3 of the 33 (9%) cytological and 8 of the 39 (21%) histological specimens. In total, *HER2* amplification was present in 11 of the 72 (15%) metastases. Two of the three *HER2*-amplified cytological samples (one retroperitoneal lymph node and one liver metastasis) showed a *HER2* cluster pattern in the whole-tumour cell population, whereas the third sample, a sovraclavear lymph node metastasis, had an average gene copy number of 12 and a *HER2*/*CEP17* FISH ratio of 4.0 in 90% of tumour cells. All three amplified metastatic lesions sampled by cytology were synchronous. A cluster pattern of amplification was present in the eight *HER2*-positive metastases sampled by core tissue biopsy (seven liver and one pancreatic lesions); seven of these were synchronous and one metachronous.

Of the 61 *HER2*-negative metastases, 28 (46%) were disomic and 33 (54%) polysomic. No difference was observed in the distribution of *HER2* gene copy number, between FNAB samples (*n*=26; mean value=3.29) and core biopsy or surgical resection samples (*n*=31; mean value=2.96) (independent samples *t*-test=NS).

The *HER2* amplification was observed in 10 of the 68 (15%) primary tumours (Table 2). Among them, nine cases showed a cluster amplification in >90% of tumour cells population, whereas one case showed intra-tumour heterogeneity, having a different *HER2* asset in different areas of the tumour: 33% of tumour cells had an average *HER2* gene copy number of 4.5 and a *HER2*/*CEP17* ratio of 2.4, whereas polysomy was present in 67% of nuclei with an average *HER2* gene copy number of 3.6 and a *HER2*/*CEP17* FISH ratio of 1.27. In all, 34 (59%) of the 58 unamplified primary tumours were disomic, and 24 (41%) polysomic. A *r*^2^ of 0.937 and of 0.962 was found for FISH count by two independent observers in primary and metastatic lesions, respectively.

**Figure 1:**
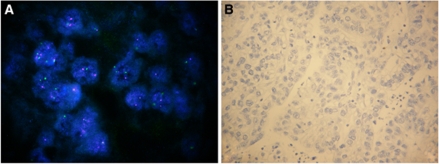
Gastric carcinoma with neither HER2 amplification (**A**) nor protein overexpression (**B**) in the primary tumour. Original magnification: (**A**) × 1250; (**B**) × 400.

**Figure 2:**
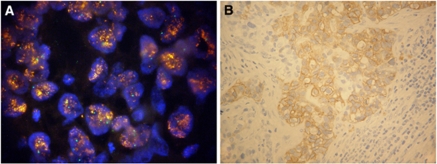
The HER2 cluster amplification (**A**) and protein overexpression (**B**) in the lymph node metastasis of the primary gastric carcinoma shown in Figure 1. Original magnification: (**A**) × 1250; (**B**) × 400.

Among the 68 cases assayed by FISH on both primary and paired metastatic site, 10 cases were amplified in both specimens (Table 2). In all, 9 of the 10 cases showed a cluster amplification in both primary and metastasis; the tumour with heterogeneous *HER2* amplification in the primary showed an increase in both *HER2* gene copy number (4.5 *vs* 12.0) and the percentage of amplified cells (33 *vs* 90%) in the matched metastasis. A single case showed primary *vs* metastasis discordance, being unamplified in the primary tumour (Figure 1A) and exhibiting a cluster amplification in 20% of tumour cells in the matched synchronous pancreatic lymph node biopsy (Figure 2A).

The HER2 protein overexpression was assessed by IHC on histological sections obtained from the 72 primary tumours and the 39 metastatic lesions sampled by core biopsies or surgical resection. In all, 10% (7/68) of the cases resulted positive by IHC, 6 of them showing a immunopositive (3+) reaction in >80% of tumour cells and 1 in 50% of tumour cells. The total concordance between IHC and FISH was 95.6%. FISH and IHC results in the 68 comparable primary tumours are shown in Table 3.

As to the 39 metastasis specimens assessed by IHC, 22 cases were negative by both IHC and FISH, 7 were positive by both techniques and 10 cases were ICH equivocal, 9 of which resulted FISH negative (Table 4). Table 5 shows the comparison between HER2 protein expression evaluated on histological sections of 39 primary tumours and paired metastatic sites. Overall concordance was 94.9%. Of the 39 matched cases the single discordant case was that also showing discordant FISH results. In this case, HER2 immunostaining was negative in the primary tumour (Figure 1B), whereas it showed a strong basolateral staining in 15% of cells in the metastasis (Figure 2B).

## Discussion

The HER2 status in gastric carcinoma can be evaluated by IHC or FISH technique and is usually assessed on the primary tumour because patients with recurrent disease rarely undergo surgery or biopsy. The issue of concordance of *HER2* status between primary tumour and metastasis has been scarcely addressed in the scientific literature. Only one study (Marx *et al*, 2009) has been previously published on this subject. The authors reported an identical *HER2* status, as assessed by FISH, in 49 primary tumours and their corresponding lymph node metastases.

Our results confirmed a high level of concordance between IHC and FISH methodologies to assess HER2 status in gastric cancer, as previously reported by other authors (Takehana *et al*, 2002; Yano *et al*, 2006; Hofmann *et al*, 2008; Marx *et al*, 2009).

The high concordance observed between IHC and FISH results on primaries and corresponding metastases, points out that in gastric cancer HER2 status is maintained in most cases unchanged during the metastatic process. However, given the low number of cases analysed, at the present time we cannot draw any conclusion about HER2 asset in synchronous and metachronous metastases.

The *HER2* amplification was observed in 21% of the metastatic lesions sampled by histology and in 9% of those sampled by cytology. This difference cannot be ascribed to a bias of cytology given that FISH results were entirely concordant with those obtained on the histological specimens of the corresponding primary tumours. It is likely that the discrepancy observed between the rate of cases scored as positive on cytology and on histology could depend on the small sample size deserving further validation in a larger cohort of patients. However, among cytological cases, one tumour *HER2* amplified on both the primary and the metastasis, was discordant by IHC.

The *HER2* amplification status was evaluable by FISH in 68 paired primary and metastatic lesions: 10 were amplified and 57 unamplified in both sites. Only one discordant case was found, being unamplified in the primary tumour and showing a cluster amplification in 20% of tumour cells in the matched pancreatic lymph node metastasis. This result was confirmed by IHC. Among cases amplified in both the primary and the metastatic lesion, one primary tumour showed the coexistence of clones with a low level of *HER2* amplification in the context of a prevalent polysomic cell population. The percentage of amplified cells increased from 33 to 90% in the paired metastasis sampled by cytology, with a gain in both *HER2* gene copy number (12.0 *vs* 4.5) and *HER2*/*CEP17* FISH ratio (4.0 *vs* 2.4). The case was also HER2 negative by IHC on the primary; unfortunately, HER2 expression was not evaluable on the metastasis that was sampled by cytology.

The FISH is considered negative for *HER2* amplification if gene copy number is <4 and equivocal when the number is 4.0 to 6.0 following the American Society of Clinical Oncology/College of American Pathologist guideline recommendations (Shah *et al*, 2010). On the basis of these criteria that were elaborated in breast cancer, the latter case should be considered discrepant as HER2 was equivocal in the primary tumour, whereas amplification was found in the metastasis. As intratumoural heterogeneity of *HER2* amplification is more common in the stomach than in the breast (Hofmann *et al*, 2008), validation of current criteria might be warranted for gastric cancer. The case also suggests potential mechanisms to explain discrepancy. In fact, at least part of the discrepant cases might arise by a clonal selection of *HER2*-positive cells over disease progression. The higher mean of *HER2* gene copy number observed in cytological samples of metastases compared with primary tumours is also better explained by enrichment of *HER2*-positive cells from a starting heterogeneous tissue rather than by technical issues such as sampling or FISH evaluation of intact *vs* truncated nuclei.

As concerns the other cases defined as positive by FISH, we observed that amplification was present within the whole-tumour cell population; on the other hand a immunopositive reaction (3+) was present at least in 50% of tumour cells in all the cases scored as positive by IHC.

Another question, still matter of debate in breast cancer, concerns the clinical significance of Chr17 polysomy on survival and response to trastuzumab therapy (Rosenberg, 2008). Compared with breast cancer in which <10% of tumours shows increased *CEP17* copy number (Shah *et al*, 2010), we observed a prevalence of 35.3% (24/68) of Chr17 polysomy in our series, an observation that underscores the need of further investigations on the predictive and prognostic significance of Chr17 polysomy in gastric carcinoma.

The results of the phase III ToGA trial (Bang *et al*, 2010) indicate trastuzumab plus cisplatin and fluoropyrimidine chemotherapy as the standard of care for patients with HER2-positive advanced gastric cancers. In this setting, the present findings are clinically relevant as they confirm current clinical practices in the assessment of HER2 status. Nonetheless, the evidence of two HER2-positive metastasic lesions with one negative and one equivocal primaries suggest that HER2 reassessment of the metastasis might be considered in selected cases when this would change the therapeutic decision.

## Figures and Tables

**Table 1:** Patient characteristics

	**No. of patients (*n*=72)**	**%**
*Gender*		
Male	50	69
Female	22	31
		
Age, range	49–88	
		
*Type of intervention*		
Surgical resection	58	81
Biopsy	14	19
		
*Primary tumour site*		
Gastroesophageal junction	6	8
Other	66	92
		
*Histology*		
Lauren classification		
Diffuse	29	40
Intestinal	33	46
Mixed	6	8
Not classified	4	6
		
*Metastatic sites*		
Liver	19	26
Pleural fluid	4	6
Ascitic fluid	11	15
Skin	4	6
Lymph nodes		
Regional	2	3
Distant	14	19
Peritoneum	8	11
Other sites	10	14
		
Synchronous metastases	36	50
Metachronous metastases	36	50

**Table 2:** *HER2* FISH on distant metastatic sites of gastric carcinoma and matched histological specimens from the primary tumours

	***HER2* distant metastatic sites**	
	**FISH−**	**FISH+**
*HER2 primary tumours*		
FISH−	57	1
FISH+	0	10

Abbreviations: FISH=fluorescence *in situ* hybridisation; HER2=human epidermal growth factor receptor 2.

*n*=68.

Concordance=98.5%.

**Table 3:** HER2 status assessed by FISH and IHC in 68 primary gastric carcinomas

	**IHC score**			
	**Negative**		**Equivocal**	**Positive**
	**0**	**1+**	**(2+)**	**(3+)**
FISH+	1	0	2	7
FISH−	44	7	7	
Total	45	7	9	7

Abbreviations: FISH=fluorescence *in situ* hybridisation; HER2=human epidermal growth factor receptor 2; IHC=immunohistochemistry.

Concordance: 95.6%.

**Table 4:** Comparison of HER2 status assessed by both IHC and FISH on 39 histological sections obtained from metastatic sites of gastric carcinoma

	**FISH**	
	**Negative, *n* (%)**	**Positive, *n* (%)**
*IHC score*		
Negative		
0	12	
1+	10	
Equivocal (2+)	9	1
Positive (3+)		7

Abbreviations: FISH=fluorescence *in situ* hybridisation; HER2=human epidermal growth factor receptor 2; IHC=immunohistochemistry.

*n*=39.

Pearsons's *R*=0.829, *P*<0.001.

**Table 5:** Comparison of HER2 status assessed by IHC on 39 primary gastric carcinomas and corresponding metastatic sites

	**IHC metastatic sites**			
	**Negative**		**Equivocal (2+)**	**Positive (3+)**
	**0**	**1+**		
*IHC primary tumours*				
Negative				
0	12	3	2	1
1+		6	1	
Equivocal (2+)		1	7	1
Positive (3+)				5

Abbreviations: HER2=human epidermal growth factor receptor 2; IHC=immunohistochemistry.

*n*=39.

*κ*=0.723, *P*<0.001.
