# Comparison of HER2 status in primary and paired metastatic sites of gastric carcinoma

**DOI:** 10.1038/bjc.2011.208

**Published:** 2011-06-07

**Authors:** C Bozzetti, F V Negri, C A Lagrasta, P Crafa, C Bassano, I Tamagnini, G Gardini, R Nizzoli, F Leonardi, D Gasparro, R Camisa, S Cavalli, E M Silini, A Ardizzoni

**Correction to**: *British Journal of Cancer* (2011) **104**, 1372–1376; doi:10.1038/bjc.2011.121

Upon publication earlier in Volume 104, the corresponding author noted that one of the co-authors’ names had been incorrectly spelled. S Cavalli had instead been presented as ‘S Capelli’. The authors and publishers are now happy to correct this mistake – the full, correct authorship is shown above.

